# Interobserver reliability of the Tile classification system for pelvic fractures among radiologists and surgeons

**DOI:** 10.1007/s00330-020-07247-0

**Published:** 2020-09-08

**Authors:** Tobias Zingg, Emilie Uldry, Patrick Omoumi, Daniel Clerc, Arnaud Monier, Basile Pache, Mohammed Moshebah, Fabio Butti, Fabio Becce

**Affiliations:** 1 Department of Visceral Surgery, Lausanne University Hospital and University of Lausanne, Lausanne, Switzerland; 2 Department of Diagnostic and Interventional Radiology, Lausanne University Hospital and University of Lausanne, Lausanne, Switzerland

**Keywords:** Reproducibility of results, Pelvic fractures, Multidetector computed tomography, Radiologists, Surgeons

## Abstract

**Objectives:**

To assess the interobserver reliability (IOR) of the Tile classification system, and its potential influence on outcomes, for the interpretation of CT images of pelvic fractures by radiologists and surgeons.

**Methods:**

Retrospective data (1/2008–12/2016) from 238 patients with pelvic fractures were analyzed. Mean patient age was 44 years (SD 20); 66% were male. There were 54 Tile A, 82 Tile B, and 102 Tile C type injuries. The 30-day mortality rate was 15% (36/238). Six observers, three radiologists, and three surgeons with different levels of experience (attending/resident/intern) classified each fracture into one of the 26 second-order subcategories of the Tile classification. Weighted kappa coefficients were used to assess the IORs for the three main categories and nine first-order subcategories.

**Results:**

The overall IORs of the Tile system for the main categories and first-order subcategories were moderate (kappa = 0.44) and fair (kappa = 0.31), respectively. IOR was fair to moderate among radiologists, but only fair among surgeons. By level of training, IOR was moderate between attendings and between residents, whereas it was only fair between interns. IOR was moderate to substantial (kappa = 0.56–0.70) between the radiology attending and resident. Association of the Tile fracture type with 30-day mortality was present based on two out of six observer ratings.

**Conclusions:**

The overall IOR of the Tile classification system is only fair to moderate, increases with the level of rater experience and is better among radiologists than surgeons. In the light of these findings, results from studies using this classification system must be interpreted cautiously.

**Key Points:**

• *The overall interobserver reliability of the Tile pelvic fracture classification is only fair to moderate.*

• *Interobserver reliability increases with observer experience and radiologists have higher kappa coefficients than surgeons.*

• *Interobserver reliability has an impact on the association of the Tile classification system with mortality in two out of six cases.*

**Electronic supplementary material:**

The online version of this article (10.1007/s00330-020-07247-0) contains supplementary material, which is available to authorized users.

## Introduction

Pelvic ring fractures account for approximately 3% of skeletal injuries, with a reported incidence of 23/100,000 persons/year [1, 2]. They result from high-energy impacts and are usually associated with multiple injuries [3, 4]. Due to significant retroperitoneal bleeding or severe extra-pelvic injuries, most often of the chest or the central nervous system, the mortality rate from pelvic ring injuries may reach up to 30%, especially in hemodynamically unstable patients [5]. Potential pelvic bleeding sources include bony fracture surfaces, the disrupted pelvic venous plexus, and arterial bleeding from branches of iliac vessels [6, 7].

With the aim of guiding clinical management and providing a common language for both clinicians and researchers based on the fracture pattern, several classification systems for pelvic ring injuries have been developed [8–13]. One of the most frequently used is the Tile classification, first proposed in 1980 [14, 15]. It is based on the mode of mechanical pelvic ring instability. Type A fractures do not concern the pelvic ring per se and are stable, type B are rotationally unstable, whereas type C are in addition vertically unstable. Each main fracture type is further subdivided into nine first-order subcategories and a total of 26 second-order subcategories [16]. Although originally designed for use with plain radiographs, the Tile classification is now routinely used based on CT images [17], which allow for a more precise evaluation of the posterior elements of the pelvic ring [18–20] and can further aid in identifying bleeding from pelvic sources and associated abdominal injuries [21–23].

For any classification system to be useful, there should be a high interobserver reliability (IOR); otherwise, it may not be possible to correctly interpret study results or justify clinical decisions and management algorithms based on such classification systems. For instance, there is controversy about the clinical usefulness of classification systems in terms of the association of fracture patterns with the risk of significant bleeding and mortality, whether the Tile [24–30] or other systems [9, 29, 31–34] are used. So far, four studies have examined the IOR of the Tile and other classification systems, finding IORs ranging from poor to moderate. None of these studies included radiologists as observers [35–38].

The primary aim of this study was to examine the IOR of the Tile classification based on CT scans read by radiologists and surgeons with varying levels of experience. The secondary aim was to assess whether the classification ratings by different observers influenced the association between the pelvic fracture type and mortality in the present study.

## Materials and methods

### Patient selection and study design

All patients with a diagnosis of pelvic fracture and CT images obtained in the emergency department of our tertiary referral hospital (*n* = 229) or a transferring institution (*n* = 13) during the study period from January 2008 to December 2016 were identified in the institutional trauma registry (*n* = 242). After review, patients with isolated acetabular fractures (*n* = 4) were excluded. For each case (*n* = 238), the following variables were extracted from the registry: age, gender, injury mechanism, presence of a pelvic circumferential compression device (PCCD) on arrival, Injury Severity Score (ISS), Abbreviated Injury Scale (AIS) score for head/neck, chest, abdomen and extremities/pelvis body regions, base excess (BE), lactate, systolic blood pressure (SBP), heart rate (HR), intensive care unit (ICU), length of stay (LOS), interventions (surgery, arterial angio-embolization), and 30-day mortality.

The present manuscript was prepared to conform to the Strengthening the Reporting of Observational Studies in Epidemiology (STROBE) guidelines [39] and the study protocol was approved by the local institutional review board (Protocol number 2016-927).

### CT protocol

For the vast majority of cases (*n* = 226, 95%), the institutional whole-body trauma CT protocol was performed using a 64- or 256-detector row CT scanner (LightSpeed VCT and Revolution CT; GE Healthcare) on arrival in the emergency radiology department. Relevant standardized pelvic CT data acquisition settings were as follows: tube potential, 120 kVp; tube current, ~ 400 mA; gantry revolution time, 0.5–0.6; beam collimation, 64 or 128 × 0.625 mm; and pitch, 0.992–1.375. Pelvic CT images were reconstructed at a section thickness/interval of 1.25/1 mm using both smooth (standard) and sharp (bone) kernels and iterative reconstruction algorithms (ASiR and ASiR-V, GE Healthcare; from 2010 and 2015 onwards, respectively).

### Image analysis

In order to define the diagnostic reference values, two experts, one musculoskeletal radiologist (13 years of experience) and one emergency general surgeon (16 years of experience), independently reviewed all CT scans and attributed a second-order subcategory fracture type according to the Tile classification system to each case. When there was disagreement, CT images were independently reviewed by a third expert, an orthopedic trauma surgeon (17 years of experience), with adjudication and final consensual decision in a joint session.

CT scans were then independently reviewed by six other observers, three radiologists and three surgeons, who were blinded to patient characteristics, treatments, outcomes, and the classification ratings of their peers. Each specialty was represented by an attending (radiologist, 15 years; surgeon, 14 years of experience), a resident (radiologist, 6 years; surgeon, 7 years of experience), and a first-year intern. The observers were all provided with the same description of the Tile classification system [16] prior to reviewing the whole CT image datasets (axial images with coronal and sagittal reformations, with the availability of 2D oblique sections and 3D reconstructions using the multiplanar reformation and volume rendering view modes, respectively) using a picture archiving and communication system (Vue, Carestream Health), without any time constraints. Fifty cases were randomly chosen and reviewed by six other peers (three from each specialty) with comparable levels of experience (two attendings with 13 and 16 years, two residents with 5 years each, and two interns in their first clinical year) in order to check for internal consistency.

### Statistical analysis

For categorical variables, results were expressed in frequencies and percentages. For continuous variables, a measure of dispersion was given using medians with interquartile ranges (IQR) for data with a skewed distribution or means with standard deviation (SD) for normally distributed data. Associations between categorical variables and binary outcomes were evaluated using Pearson’s chi-squared test. Weighted kappa coefficients were used to measure IOR, which were interpreted according to Landis and Koch. Kappa values of 0–0.20 indicate poor, 0.21–0.40 fair, 0.41–0.60 moderate, 0.61–0.80 substantial, and 0.80–1 almost perfect agreement [40]. Since the minimal number of observations (*k*) for valid kappa statistics is (2 × *k*^2^) [41], only IORs for the main (*k* = 3, *n* = 18) and first-order (*k* = 9, *n* = 162) subcategories were analyzed. Cohen’s kappa [42] was used when comparing two raters and Fleiss’s kappa [43] for combined kappa values of three or more raters. All analyses were performed using Stata/IC v15.1 (StataCorp LLC). The distribution of kappa weights is illustrated in Supplementary Table 1. A significance threshold with a two-sided *p* value of 0.05 was adopted for all statistical analyses.

## Results

**Fig. 1:**
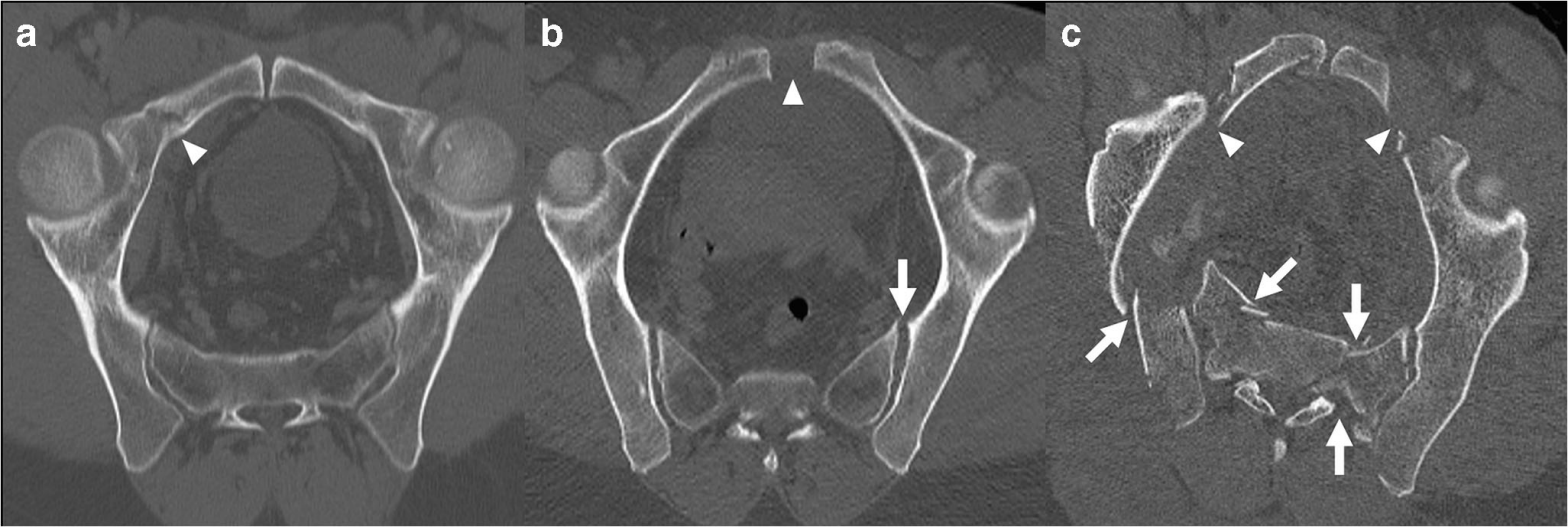
Representative axial-oblique reformatted CT images showing the pelvic ring of patients with Tile A (2.2) (**a**), Tile B (1.1) (**b**), and Tile C (3.3) (**c**) fracture types. Arrowheads show disruption of the anterior arch, while arrows indicate disruption of the posterior arch of the pelvic ring. In Tile A fractures, the posterior arch is spared, while it is partially disrupted in Tile B and completely disrupted in Tile C fracture types

Among the 238 pelvic fractures included for analysis, there were 54 Tile A (23%), 82 Tile B (34%), and 102 (43%) Tile C types. In the present study cohort, the mean ISS was 24 (SD, 13) and the most frequently associated major (AIS ≥ 3) injuries concerned the chest in 98 (41%), the abdomen in 54 (23%), and the head in 53 (22%) patients. Mean patient age was 44 years (SD, 20 years); 158 patients (66%) were males. Surgical stabilization of the pelvis was performed in 52 (22%) of patients and 25 (11%) underwent arterial angio-embolization for active pelvic bleeding. Table 1 summarizes the detailed diagnostic reference values based on the consensual review by the three experts, and Table 2 shows the characteristics of the study population. Figure 1 illustrates examples of the three main categories (A/B/C) of the Tile classification.

**Table 1:** Reference Tile classifications of pelvic fractures (*n* = 238)

Categories, *n* (%)		
Main	1st order	2nd order
A: 54 (23)		
	A1: 2 (0.8)	
		A1.1: 1 (0.4)
		A1.2: 1 (0.4)
	A2: 28 (16)	
		A2.1: 16 (7)
		A2.2: 17 (7)
		A2.3: 5 (2)
	A3: 14 (6)	
		A3.1: 1 (0.4)
		A3.2: 8 (3)
		A3.3: 5 (2)
B: 82 (34)		
	B1: 7 (3)	
		B1.1: 5 (2)
		B1.2: 2 (0.8)
	B2: 67 (28)	
		B2.1: 52 (22)
		B2.2: 14 (6)
		B2.3: 1 (0.4)
	B3: 8 (3)	
		B3.1: 1 (0.4)
		B3.3: 7 (3)
C: 102 (43)		
	C1: 60 (25)	
		C1.1: 1 (0.4)
		C1.2: 23 (10)
		C1.3: 36 (15)
	C2: 17 (7)	
		C2.1: 1 (0.4)
		C2.2: 9 (4)
		C2.3: 7 (3)
	C3: 25 (11)	
		C3.1: 4 (2)
		C3.2: 2 (0.8)
		C3.3: 19 (8)

**Table 2:** Demographics and characteristics of the study population (*n* = 238)

*n* (%)	238 (100)
Age (years), mean (SD)	44 (20)
Male gender, *n* (%)	158 (66)
ISS, mean (SD)	24 (13)
AIS head/neck, mean (SD)	1.3 (1.7)
AIS chest, mean (SD)	1.8 (1.5)
AIS abdomen, mean (SD)	1.5 (1.4)
AIS extremities/pelvis, mean (SD)	3.1 (1)
Admission SBP (mmHg), mean (SD)	125 (27)
Admission HR (BPM), mean (SD)	95 (22)
Base excess (mEq/l), median (IQR)	-3.9 (-1.9 to -7.4)
Lactate (mmol/l), median (IQR)	2.3 (1.4–3.9)
Prehospital PCCD placed, *n* (%)	151 (63)
Surgical pelvic stabilization, *n* (%)	52 (22)
External fixation, *n* (%)	16 (6.7)
Primary ORIF, *n* (%)	18 (7.6)
External fixation followed by ORIF, *n* (%)	18 (7.6)
Arterial angio-embolization for pelvic bleeding, *n* (%)	25 (11)
ICU LOS (days), median (IQR)	0 (0–3)
30-day mortality, *n* (%)	36 (15)
Injury mechanism:	
Falls, *n* (%)	112 (47)
Road traffic accidents	
Cyclist, *n* (%)	12 (5)
Motor vehicle, *n* (%)	73 (31)
Pedestrian hit, *n* (%)	28 (12)
Crush, *n* (%)	11 (5)
Other, *n* (%)	2 (0.8)

*AIS* abbreviated injury scale, *BPM* beats per minute, *HR* heart rate, *ICU* intensive care unit, *IQR* interquartile range, *ISS* injury severity score, *LOS* length of stay, *ORIF* open reduction internal fixation, *PCCD* pelvic circumferential compression device, *SD* standard deviation, *SBP* systolic blood pressure

For the three main Tile categories (A, B, C), the combined IOR for all six observers was moderate (kappa = 0.44). When analyzed by specialty, IOR was moderate among radiologists (kappa = 0.47) but only fair among surgeons (kappa = 0.34). The combined IOR for the nine first-order subcategories (A1–3, B1–3, C1–3) was fair, overall (kappa = 0.31) and by specialty, yet kappa values were higher among radiologists (kappa = 0.35) than among surgeons (kappa = 0.23). Table 3 summarizes the combined IORs with the corresponding kappa values.

**Table 3:** Combined interobserver reliabilities

	Kappa^§^	*p*
Tile main categories (A, B, C)		
All, 6 observers	0.4410	< 0.001
RAD, 3 observers	0.4731	< 0.001
SURG, 3 observers	0.3435	< 0.001
Tile first-order subcategories (A1–3, B1–3, C1–3)		
All, 6 observers	0.3123	< 0.001
RAD, 3 observers	0.3525	< 0.001
SURG, 3 observers	0.2278	< 0.001

*RAD* radiology, *SURG* surgery

^§^Fleiss’s kappa

For both main and first-order subcategories, the individual two-rater IORs between each of the six observer ratings and the reference classification were substantial for the two attendings (radiology and surgery; kappa = 0.79 and 0.74, respectively) and the radiology resident (kappa = 0.79). They were moderate for the surgery resident (kappa = 0.58) and interns of both specialties (kappa = 0.43 and 0.52, respectively).

By general level of experience, the two-rater IORs were moderate for attendings (kappa = 0.60 and 0.41) and residents (kappa = 0.55 and 0.42), but only fair for interns (kappa = 0.33 and 0.27), both for the main and first-order subcategories. Kappa values were consistently higher for the main categories.

By level of experience and specialty, the two-rater IORs were only fair whenever one of the raters was an intern, both for the main (kappa = 0.37–0.40) and first-order subcategories (kappa = 0.28–0.37). Again, kappa values were consistently higher for the main categories. For the attending-resident pairs, the IORs were substantial for radiology (kappa = 0.70) and moderate for surgery (kappa = 0.47) for the main categories but decreased to moderate (kappa = 0.56) and fair (kappa = 0.31), respectively, for the first-order subcategories. Table 4 summarizes all two-rater kappa values.

**Table 4:** Two-rater interobserver reliabilities

	Observed agreement (%)	Expected agreement (%)	Kappa^§^	Standard error	*p*
Tile main categories (A, B, C)					
REF—RAD attending	87.92	41.46	0.7937	0.0454	< 0.001
REF—RAD resident	87.71	42.71	0.7855	0.0459	< 0.001
REF—RAD intern	63.97	36.91	0.4289	0.0428	< 0.001
REF—SURG attending	85.29	43.47	0.7399	0.0459	< 0.001
REF—SURG resident	76.05	43.41	0.5768	0.0440	< 0.001
REF—SURG intern	71.53	40.37	0.5226	0.0429	< 0.001
RAD—SURG attendings	76.58	41.93	0.5966	0.0451	< 0.001
RAD—SURG residents	74.58	44.09	0.5454	0.0449	< 0.001
RAD—SURG interns	56.83	35.76	0.3280	0.0416	< 0.001
RAD attending—resident	82.56	41.18	0.7036	0.0452	< 0.001
RAD intern—attending	61.97	36.75	0.3988	0.0435	< 0.001
RAD resident—intern	62.39	37.21	0.4011	0.0433	< 0.001
SURG attending—resident	70.69	44.26	0.4742	0.0438	< 0.001
SURG intern—attending	64.60	40.63	0.4038	0.0423	< 0.001
SURG resident—intern	58.93	35.26	0.3656	0.0363	< 0.001
Tile first-order subcategories (A1–3, B1–3, C1–3)					
REF—RAD attending	76.55	23.61	0.6931	0.0296	< 0.001
REF—RAD resident	74.08	23.77	0.6599	0.0299	< 0.001
REF—RAD intern	52.73	18.96	0.4167	0.0259	< 0.001
REF—SURG attending	70.55	23.33	0.6158	0.0291	<0.001
REF—SURG resident	57.23	21.58	0.4546	0.0271	< 0.001
REF—SURG intern	58.03	24.50	0.4441	0.0288	< 0.001
RAD—SURG attendings	54.62	22.95	0.4111	0.0283	< 0.001
RAD—SURG residents	54.75	22.55	0.4157	0.0279	< 0.001
RAD—SURG interns	40.38	18.34	0.2699	0.0240	< 0.001
RAD attending—resident	65.84	22.97	0.5566	0.0287	< 0.001
RAD intern—attending	46.60	18.41	0.3455	0.0252	< 0.001
RAD resident—intern	49.79	19.96	0.3727	0.0269	< 0.001
SURG attending—resident	45.97	21.74	0.3096	0.0270	< 0.001
SURG intern—attending	48.45	23.60	0.3252	0.0275	< 0.001
SURG resident—intern	41.39	18.33	0.2823	0.0231	< 0.001

*RAD* Radiology, *REF* Reference classification, *SURG* Surgery

^§^Cohen’s kappa

The agreement on the 50 randomly chosen control cases between the designated raters and their peers with a comparable level of expertise ranged from moderate to almost perfect (kappa = 0.57–0.81) (Supplementary Table 2).

The overall 30-day mortality rate of the study cohort was 15% (36/238). Based on the reference classification by the three experts, none of the main Tile categories (A, B, C) of pelvic fractures was associated with mortality (*p* = 0.06). However, when based on the classification ratings of each of the six observers separately, an association of Tile C fracture types with mortality was observed for two out of six raters. Table 5 summarizes the association of main Tile categories of pelvic fractures with 30-day mortality for the reference and each rater.

**Table 5:** 30-day mortality by Tile main pelvic fracture categories and observers

	Tile A, *n* (%)			Tile B, *n* (%)			Tile C, *n* (%)			*p*^§^
	All	Alive	Dead	All	Alive	Dead	All	Alive	Dead	
Reference	54	48 (89)	6 (11)	82	74 (90)	8 (10)	102	80 (78)	22 (22)	0.06
Observer 1	48	43 (90)	5 (10)	83	76 (92)	7 (8)	107	83 (78)	24 (22)	0.02
Observer 2	59	54 (92)	5 (8)	35	31 (89)	4 (11)	144	117 (81)	27 (19)	0.14
Observer 3	64	57 (89)	7 (11)	127	106 (83)	21 (17)	47	39 (83)	8 (17)	0.55
Observer 4	62	55 (89)	7 (11)	95	82 (86)	13 (14)	81	65 (80)	16 (20)	0.33
Observer 5	56	49 (88)	7 (12)	75	64 (85)	11 (15)	107	89 (83)	18 (17)	0.76
Observer 6	109	96 (88)	13 (12)	52	48 (92)	4 (8)	77	58 (75)	19 (25)	0.01

^§^Pearson’s chi-squared test was used to measure the association between mortality and Tile categories (A, B, C)

## Discussion

The results of this study show that the overall combined IORs for the Tile pelvic fracture classification system using CT alone are moderate for the three main categories and fair for the nine first-order subcategories. Radiologists had a higher combined IOR (moderate) than surgeons (fair), but only for the three main categories. The IORs of the attendings from both specialties and the radiology resident were substantial, but only moderate for the other raters. These findings suggest an improvement of IOR with increasing level of experience and radiological specialization. When taking the classifications by each rater individually and relating them to mortality, a significant association was observed in two out of six cases, showing the potential influence of IOR on study results, conclusions, and potential implications for clinical decision-making, management algorithms, and recommendations.

For any imaging-based classification system to be useful, there should be a high level of inter- and intraobserver agreement. This has important implications for communication in research (comparability of study results) and, in consequence, clinical activities (classification-based outcome prediction or management guidelines). The importance of reliably linking imaging findings with outcomes in musculoskeletal radiology studies has been recently highlighted by Tagliafico et al [44]. Several classification systems for pelvic fractures have been proposed in the past [9, 11–15, 18]. Only four studies [35–38] assessing the IORs of the Tile [14, 15] and other classification systems [13, 18] have been published so far. To our knowledge, the present study is the largest to date on the IOR of the Tile pelvic fracture classification, allowing for a statistically sound evaluation of the first-order subcategory. It is also the first to include not only surgeons but also radiologists with different levels of experience among the raters.

Koo et al [35] were the first to evaluate the Tile and the Young-Burgess (YB) [18] classification systems by assessing IORs based on the interpretation of plain radiographs first and then comparing them with IORs based on the ratings of CT scans. All six observers were orthopedic traumatologists with different levels of expertise (pelvic/acetabular specialists, orthopedic traumatologists, and orthopedic trainees). Although only 30 patients were included in their study, subcategories were also assessed and results reported. The IORs for the Tile system pre- (kappa = 0.30) and post-CT (kappa = 0.33) were only fair, mainly due to the poor agreement among raters who were not pelvic/acetabular specialists. In line with our findings, advanced observer experience increased the IOR (from kappa = 0.07 to kappa = 0.84). One of the main clinically relevant findings in their study was that CT, compared with plain radiographs alone, improved the reliability of fracture stability assessment from moderate to almost perfect. Furey et al [36], in addition to IOR, also assessed the intraobserver reliability of both the Tile and YB systems. Their study, conducted at the center where the YB classification was designed, was based on plain radiographs and CTs for each fracture and included 89 patients and 5 observers (all experienced orthopedic trauma surgeons). Only the three main Tile categories were evaluated. Moderate IORs were found for both the Tile (kappa = 0.47) and YB (kappa = 0.46) classifications. Intraobserver reliabilities were only moderate for the Tile (kappa = 0.47), but substantial for the YB (kappa = 0.72) main classifications. Gabbe et al [37] rated 100 pre-interventional (no PCCDs or external fixators placed) pelvic CTs and plain radiographs (anteroposterior view only) by three experienced orthopedic surgeons (over 52 years of combined experience in managing pelvic fractures) from different Australian level 1 trauma centers. The authors found only slight IORs for both the Tile (kappa = 0.10–0.17) and YB (kappa = 0.17–0.19) main classifications, insufficient for clinical or research purposes according to their conclusion. Berger-Groch et al [38] recently published a study including 154 patients with pelvic fractures. Only CT images were interpreted by four observers (two senior orthopedic surgeons, one resident in training, and one medical student). Inter- and intraobserver reliabilities were assessed for the Tile, YB, and Rommens [13] classification systems. The overall IORs, expressed as intraclass correlation coefficients [45], were fair for the Tile (0.55) and the other classification systems and were strongly dependent on rater experience.

There may be several causes for the only slight to moderate overall IORs of the Tile classification system in the present and previous studies. One explanation may be the weight of disagreements among inexperienced raters, decreasing the combined IORs, despite substantial to almost perfect IORs among experienced orthopedic traumatologists. This could be observed in both studies that included raters with different levels of training, showing an improvement of IOR with increasing rater experience. However, one of the two studies with exclusively experienced raters also showed only moderate inter- and intraobserver reliabilities for the Tile classification system. Interestingly, the intraobserver reliability in that study was substantial for the YB system, potentially attributable to the fact that the raters routinely worked with the YB classification, and not with the Tile system [36]. Inter- and intraobserver reliabilities may therefore also depend on the regularity with which any system is applied by a given observer. The second study with only experienced observers found an only slight IOR of the Tile system. The presence of a greater number of more complex Tile type B and C fractures compared with the other previously published studies may explain the only slight IORs in their series [37]. However, the higher, moderate overall IORs in the present patient cohort, despite the highest rate of Type B and C fractures among all studies, do not support this hypothesis.

## Limitations and strengths

Our study has several limitations. Firstly, patients with PCCDs in place while undergoing CT (63%) were included in the present study. It has been shown previously that the presence of PCCDs may lead to misinterpretation of the fracture pattern and thus have an influence on fracture classification [46–48] and assessment of pelvic ring stability [49]. However, given the widespread use of PCCDs in the prehospital setting [50], this situation is now frequently encountered and represents clinical conditions under which fracture classifications have to occur. Secondly, as highlighted by Gabbe et al in their study [37], attributing mortality to any particular pelvic fracture type of the Tile classification based on CT is problematic, since the most severely injured patients presenting with hemodynamic instability and the highest mortality frequently do not undergo CT imaging. Therefore, the association of CT-based pelvic fracture type with mortality must be interpreted with caution. Thirdly, since it is routinely used at our center, we only assessed the Tile and no other classification system, unlike all other studies [35–38]. As suggested by the results of Furey et al [36], who observed better intraobserver reliabilities for the system routinely used at their hospital, we think there would have been a bias in the results for all not routinely used classification systems. Furthermore, unlike in three of the four existing studies [35, 36, 51], we only assessed IORs for CTs and not for plain radiographs. The current clinical practice no longer includes the three plain radiographical views for which the Tile classification was initially developed [52]. In our center, only plain radiographs (anteroposterior view) of the pelvis are obtained for hemodynamically unstable trauma patients. Hemodynamically stable trauma patients undergo a routine contrast-enhanced whole-body CT.

The present study is the first to include radiologists among the raters, and the largest so far assessing IORs of the Tile classification system, allowing for statistically appropriate evaluation of its first-order subcategories. Second-order subcategories were not assessed for IOR in the present study. A statistically appropriate analysis would have required the inclusion of 1352 cases. Given the decrease in IOR from moderate to fair from the main to the first-order subcategories, we believe that a second-order subcategory IOR below, or at best equal to fair, can be expected. Therefore, the extension of the observation period or inclusion of other centers to reach the required case number was considered excessive.

## Conclusion

Similar to the results of previous studies, the overall IOR of the Tile classification system is only fair (nine first-order subcategories) to moderate (three main categories). It depends not only on the level of experience of the observers, but it also seems to be better among radiologists than surgeons. However, even among radiologists, misclassification of pelvic fractures by the least experienced, likely to occur in emergency radiology departments and particularly during night shifts, may have important implications in clinical decision-making and management of severely injured patients. In research settings, misclassification may lead to erroneous study results and conclusions, with subsequent inappropriate translation into clinical management algorithms. This may be avoided by using only classification ratings from experienced observers.

## Electronic supplementary material

ESM 1 (DOCX 22 kb)

## Abbreviations

AIS: Abbreviated injury scale
BE: Base excess
BPM: Beats per minute
HR: Heart rate
ICU: Intensive care unit
IOR: Interobserver reliability
IQR: Interquartile range
ISS: Injury severity score
LOS: Length of stay
ORIF: Open reduction internal fixation
PCCD: Pelvic circumferential compression device
SBP: Systolic blood pressure
SD: Standard deviation
