# Supplementary material for: Interobserver reliability of the Tile classification system for pelvic fractures among radiologists and surgeons
Source: Eur Radiol. 2020 Sep 8;31(3):1517–25. doi: 10.1007/s00330-020-07247-0 (PMC7880942; doi:10.1007/s00330-020-07247-0)
Supplement: Supplementary file 1 — (DOCX 22 kb) [file 330_2020_7247_MOESM1_ESM.docx]

**Electronic Supplementary Material**

| **Supplementary Table 1.** Tile classification and weights used for kappa statistics | | | | | | | | | |
| --- | --- | --- | --- | --- | --- | --- | --- | --- | --- |
| **Main categories** | A | | | B | | | C | | |
| A | 1 | | |  | | |  | | |
| B | 0 | | | 1 | | |  | | |
| C | 0 | | | 0.25 | | | 1 | | |
| **1st order sub-categories** | A1 | A2 | A3 | B1 | B2 | B3 | C1 | C2 | C3 |
| A1 | 1 |  |  |  |  |  |  |  |  |
| A2 | 0.5 | 1 |  |  |  |  |  |  |  |
| A3 | 0 | 0 | 1 |  |  |  |  |  |  |
| B1 | 0 | 0 | 0 | 1 |  |  |  |  |  |
| B2 | 0 | 0 | 0 | 0.5 | 1 |  |  |  |  |
| B3 | 0 | 0 | 0 | 0.2 | 0.2 | 1 |  |  |  |
| C1 | 0 | 0 | 0 | 0.1 | 0.1 | 0 | 1 |  |  |
| C2 | 0 | 0 | 0 | 0.1 | 0.1 | 0.4 | 0.2 | 1 |  |
| C3 | 0 | 0 | 0 | 0 | 0 | 0.4 | 0 | 0.4 | 1 |

| **Supplementary Table 2.** Two-rater Interobserver reliabilities, peer controls (n=50) | | | | | |  |
| --- | --- | --- | --- | --- | --- | --- |
|  | Observed  Agreement (%) | Expected Agreement (%) | kappa^§^ | Standard Error | *p* | |
| **Tile main categories** | | | | | |  |
| RAD attending | 88.50 | 38.78 | **0.8122** | 0.0999 | <0.001 | |
| RAD resident | 76.50 | 39.84 | **0.6094** | 0.1000 | <0.001 | |
| RAD intern | 75.00 | 38.38 | **0.5943** | 0.1002 | <0.001 | |
|  | | | | | |  |
| SURG attending | 79.00 | 40.40 | **0.6477** | 0.0973 | <0.001 | |
| SURG resident | 80.00 | 40.05 | **0.6664** | 0.1015 | <0.001 | |
| SURG intern | 72.50 | 35.90 | **0.5710** | 0.0865 | <0.001 | |
| *RAD* Radiology, *SURG* Surgery  ^§^Cohen’s kappa | | | | | |  |
